# In vitro assessment of multipotential therapeutic importance of *Hericium erinaceus* mushroom extracts using different solvents

**DOI:** 10.1186/s40643-022-00592-6

**Published:** 2022-09-16

**Authors:** Waleed Bakry Suleiman, Reda M. Shehata, Ahmed M. Younis

**Affiliations:** 1 Botany and Microbiology Department, Faculty of Science (Boys), Al-Azhar University, The Permanent Camp St., 6th Ward, Nasr City, 11884, Cairo, Egypt; 2 The Regional Center for Mycology and Biotechnology (RCMB), Al-Azhar University, Cairo, Egypt

**Keywords:** *Hericium erinaceus*, Antioxidants, Antiviral, Anti-inflammatory, Antimicrobial, GC–MS

## Abstract

Infectious disease is one of the major threats to humans and it is the second leading cause of death worldwide. Edible mushrooms have many nutritional and medicinal values to human health. The medicinal properties of edible mushroom extract in inhibiting pathogenic microorganisms had advantages over the use of chemically synthetic antimicrobial compounds due to less unwanted side effects and can combat microbial resistance. This study hypothesized that the polarity affects the extraction quality of *Hericium erinaceus* fruiting bodies which was prepared and subsequently affects its activity as an antimicrobial against six tested microorganisms, including MRSA, and *Streptococcus mutans*, *Enterobacter cloaca*, *Salmonella typhimurium*, and *Candida lipolytica*; antiviral against Hepatitis A virus (HAV) virus; antioxidant using 2,2-diphenyl-1-picrylhydrazyl (DPPH) free radical scavenging assay; and anti-inflammatory potential. So, the mushroom was quantitatively evaluated to assess its content of flavonoids, alkaloids, tannins, saponins, carbohydrates, protein, nitrogen, and oil. The current research clarified here that aquatic extract has a significant activity as an antioxidant (IC_50_ = 53.7 µg/mL) and antiviral (IC_50_ = 24.97 µg/mL), while ethyl acetate extract showed a reasonable antimicrobial activity rather than all tested extract against tested microorganisms. Unfortunately, all extracts under investigation possess low anti-inflammatory action according to the adopting protocol. The superior results of both water and ethyl acetate extracts were later investigated by HPTLC and GC–MS for preliminary prediction of the chemical constitution of those extracts. *H. erinaceus* mushroom succeeded to establish promising antimicrobial, antiviral, and antioxidant activities while it has low anti-inflammatory activity. Both HPTLC and GC–MS could identify the chemical constituents of the mushroom crude extract.

**Graphical Abstract:**

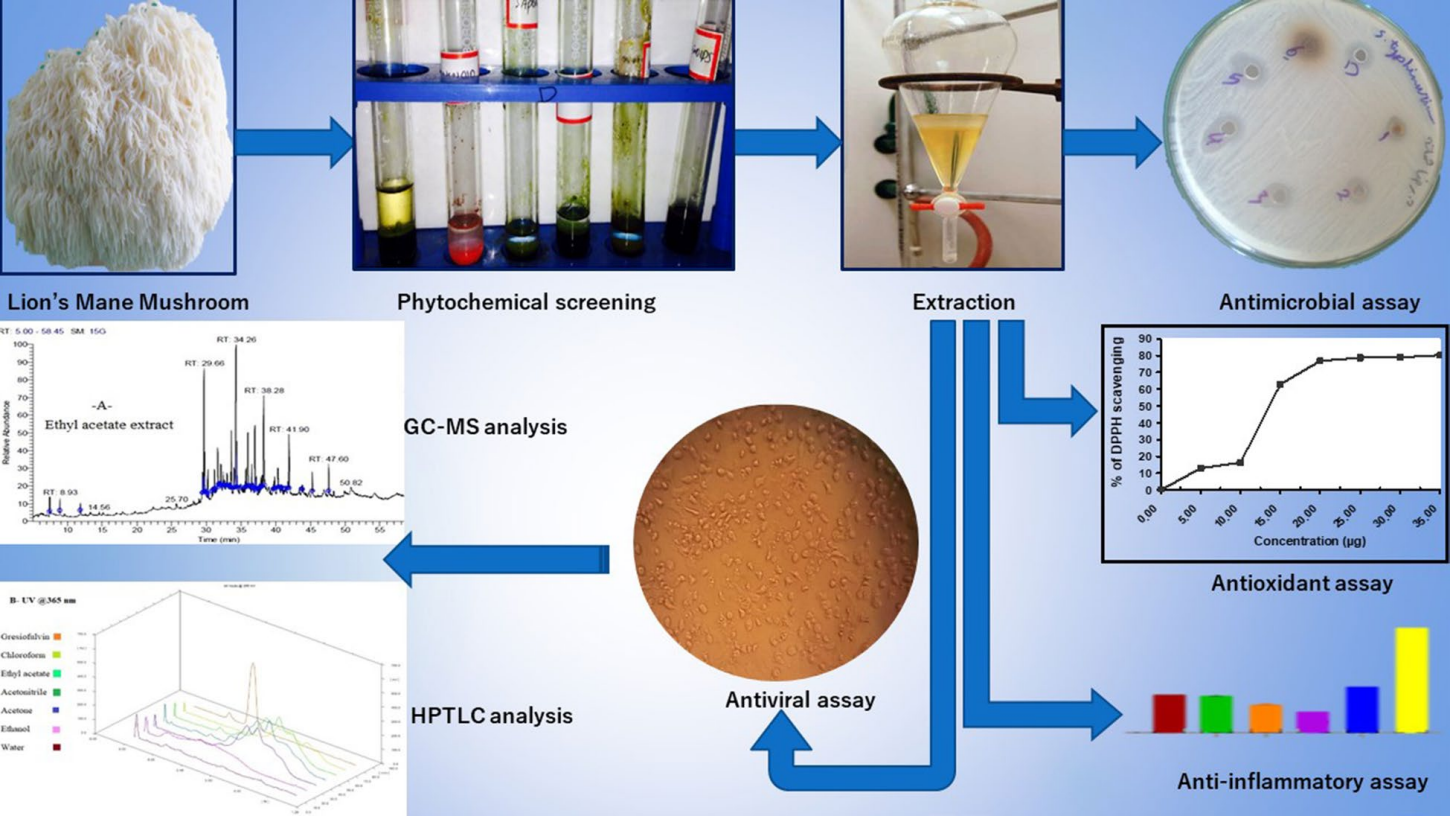

## Introduction

Infectious diseases are considered a major threat to human health, and it is the second leading cause of death worldwide. Many of these deaths occur because patients do not have access to life-saving antimicrobial compounds when and where these are needed (Nellums et al. 2018). Pathogenic bacteria contribute to other globally important diseases, such as pneumonia, tetanus, typhoid fever, diphtheria, syphilis, and leprosy foodborne illnesses, can be caused by some pathogenic bacteria, such as *Streptococcus, Clostridium, Salmonella, Corynebacterium, Treponema, Mycobacterium, Campylobacter, Shigella, Streptococcus, *and* Pseudomonas* (Wu et al. 2013; Gad et al. 2021).

Moreover, infections due to resistant bacteria are now too common and some pathogens have even become resistant to multiple types or classes of antibiotics, such as methicillin-resistant *Staphylococcus aureus* (MRSA) and vancomycin-resistant *Staphylococcus aureus* (VRSA), thus resulting in losing effective antibiotics for treatment of serious infection and can lead to death (Shawky et al. 2021). Furthermore, fungal infection generally presents more difficult therapeutic problems than bacterial infections because fungi are eukaryotes cells so compounds that can inhibit fungal growth have a higher side effect on host eukaryotic cells than antibiotics which target prokaryotic bacterial cells (Perfect 2012). In addition, fungal infections can be caused due to a long period use of antibiotics, which results in killing both pathogenic bacteria and healthy bacteria, and this alters the balance of microorganisms and results in an overgrowth of fungus (Wang et al. 2014). The continuous need for natural bioactive compounds that combat the microbial resistance to antibiotics was considered an admitted challenge, so many studies had been created to present some bioactive compounds especially from plants (Suleiman 2020) or from other sources, like microorganisms (Soliman et al. 2022).

Many mushrooms have high-quality protein content with essential amino acids and are considered a good source of vitamins, such as thiamine, riboflavin, and ascorbic acid (Cardwell et al. 2018). In the last few years, several antimicrobial and antioxidant compounds were discovered in the fungi kingdom (Sevindik 2018). Moreover, mushrooms have a wide range of secondary metabolites of high therapeutic value, such as antioxidant, diabetes, antiviral, antithrombotic, anti-inflammatory, and antitumor activities (Chaturvedi et al. 2018). Biologically active compounds isolated from mushrooms include polypeptides, polysaccharides, glycopeptides, ribonucleases, proteases, and lectins as well as low molecular weight compounds, such as lactones, terpenoids, and alkaloids (Alves et al. 2012).

In general, fungi could represent sources of several valuable compounds (Gad et al. 2022). In particular, mushrooms could be considered a good source of antioxidants that worked as protector agents against oxidative damage such as ascorbic acid and gallic acid (Ferreira et al. 2009). Also, many edible and wild mushrooms, such as *Pleurotus ostreatus* and *Laetiporus sulphureus,* showed high antimicrobial effects when extracted with polar solvents against both pathogenic bacteria and fungi (Younis et al. 2019). *Hericium erinaceus,* also known as Lion's Mane Mushroom or Hedgehog Mushroom, is an edible mushroom with historical usage in traditional Chinese medicine (Khan et al. 2013). *Hericium erinaceus* has been used traditionally and historically in East Asia to treat neurasthenia and general asthenia (Thongbai et al. 2015). It also has antitumor activities against HepG-2, MCF-7, El-4, and EC-109 (Kim et al. 2011). Additionally, *H. erinaceus* has other therapeutic uses and biological activities, such as antioxidant and anti-aging (Zhang et al. 2012), antimicrobial (Shen et al. 2015), neuroprotective activity (Zhang et al. 2016), and anti-inflammatory potential (Chong et al. 2019).

Several mushroom species had been extracted with polar and non-polar solvents and exhibited antimicrobial activities against Gram-positive and Gram-negative pathogenic bacteria (Gebreyohannes et al. 2019). Both Shiitake (*Lentinula* *edodes*) and oyster (*Pleurotus ostreatus*) mushrooms have antibacterial and antifungal properties. Moreover, extracts of the Mediterranean culinary-medicinal Oyster mushrooms *P. eryngii* var. eryngii, *P. eryngii* var. ferulae, *P. eryngii* var. elaeoselini, and *P. nebrodensis* showed growth inhibitory activity against *S. aureus* A, *S. epidermidis*, *Pseudomonas aeruginosa*, and *Escherichia coli* (Schillaci et al. 2013). In addition, several researchers reported strong antioxidant activities by mushroom extracts (Kozarski et al. 2015). Medicinal mushrooms can be a source of phenolic compounds flavonoids and polysaccharides, cytokines, and lentinan that can reduce oxidation stress (Shaffique et al. 2021). In this study, we investigated the levels of as antioxidant, anti-inflammatory, antimicrobial, and antiviral activity of *Hericium erinaceus,* in addition to forecasting the chemical constituents by GC–MS and a fully automated TLC scanner.

## Materials and methods

The fresh fruiting bodies of the edible mushrooms *H. erinaceus* named Lion's mane mushroom were collected from natural growth in Richmond, Virginia, USA. Identification was done by comparing their morphological, anatomical, and physiological characteristics and monographs with descriptions comparing their characteristics with authentic specimens present in Virginia State University herbarium (VR, USA) (Sharma 2012).

Fifty grams of air-dried fruiting bodies of *H. erinaceus* were extracted three successive times with 80% methanol, then filtered, and the combined filtrates were concentrated under reduced pressure (0.898 bar) at 50 °C using a rotary vacuum evaporator (HB4 basic, JANKE & KUNKEL IKA LABORTECHNIK) for 30 min, and then used for the following tests.

### Qualitative assessment of phytochemicals in mushroom fruiting bodies

#### Flavonoids

According to flavonoids Shinoda’s assay, the test was carried out by adding conc. hydrochloric acid dropwise to 1 mL of methanolic extract containing a fragment of magnesium ribbon where positive result gives pinkish color (Jaradat et al. 2015).

#### Alkaloids

The dried extract of *H. erinaceus* was dissolved in 2 N hydrochloric acid in a water bath, shaken, and filtered. The obtained filtrate was extracted with chloroform to remove the undesirable matters. The acidic aqueous layer was adjusted to alkaline pH with ammonia, where the liberated alkaloidal bases were extracted by chloroform and then tested by Mayer's (Balbaa et al. 1981), and Dragendorff’s reagents (Stahl 1969). The combined chloroform extract was filtered over anhydrous sodium sulfate and evaporated under vacuum (Sharma and Gupta 1994).

#### Test for saponins

According to Li et al. (2013), the dried extract of *H. erinaceus* was dissolved in 4 mL of distilled water and then filtered. The filtrate was vigorously shaken and a voluminous froth was developed, which persists for almost one hour.

#### Test for tannins

Distilled water was added to the alcohol-free extract and filtered. Ferric chloride solution was then added to the filtrate, where green or a bluish-black color is obtained in the presence of tannins (Suica-Bunghez et al. 2017). Zinc-chloro-iodine solution was prepared by adding zinc metal to hydrochloric acid until it no longer dissolved. It was then saturated with iodine and potassium iodide. Few drops of this solution were added to the alcoholic extract where the violet red color appears in the presence of tannins (Naegli’s solution).

### Quantitative assessment for phytochemical constituents

One gram of the air-dried *H. erinaceus* was extracted independently with 80% methanol three successive times. The extracts were concentrated, and the dried matter was then dissolved in 50 mL methanol. The alcoholic extracts were then completed to the volume of 100 mL by adding distilled water and used for the following determinations.

#### Total phenolics

One mL of the prepared extract was completed to the volume of 10 mL by adding distilled water; then 1 mL of Folin-Ciocalteu reagent was added. The latter mixture was shaken vigorously and after 5 min 10 mL of 70%Na_2_CO_3_ w/v was added and diluted immediately to 25 mL by adding distilled water. The latter mixture was incubated for 90 min at 25 °C. The absorbance was measured at 750 nm against the reagent used as a blank. A standard calibration plot was generated at 750 nm using known concentrations of gallic acid. The concentration of phenols in the tested samples was calculated from the calibration plot and is expressed as mg gallic acid equivalent of phenol/g of sample (Singleton and Rossi 1965).

#### Total flavonoids

One mL of the prepared extract was completed to the volume of 5 mL by adding distilled water. Immediately 0.3 mL 5% NaNO_2_ was added and the mixture was then left for 5 min. 0.3 mL 10% AlCl_3_ and 2 mL 1 M NaOH were added, respectively. The mixture was then diluted to a volume of 10 mL with distilled water and the formed pink color was measured at 550 nm against the reagent used as blank. A standard calibration plot was generated at 550 nm using known concentrations of rutin. The concentration of flavonoids in the tested samples was calculated from the calibration plot and is expressed as mg quercetin equivalent of flavonoids/g of sample (Moniruzzaman et al. 2014).

#### Total saponins

One gm of each *H. erinaceus* was dispersed in 10 mL of 20% ethanol. The suspension was heated over a hot water bath for 4 h with continuous stirring at about 55 °C. The mixture was filtered, and the residue was re-extracted with another 10 mL of 20% ethanol. The combined extracts were reduced to 2 mL over a water bath at about 90 °C. The concentrate was transferred into a 250 mL separator funnel and 5 mL of diethyl ether was added and shaken vigorously. The aqueous layer was recovered while the ether layer was discarded. The purification process was repeated. 15 mL of n-butanol was added. The combined n-butanol extracts were washed twice with 10 mL of 5% aqueous sodium chloride. The remaining solution was heated in a water bath. After evaporation, the samples were dried in the oven to a constant weight. The saponin content was calculated in percentage (Li et al. 2013).

#### Total soluble carbohydrates

##### Extraction

According to the described method by Pedras et al. (2017). One gram of *H. erinaceus* extract was put in a 100 mL capacity conical flask, to which 5 mL of 2% phenol water and 10 mL of 30% trichloroacetic acid were added. The mixture was shaken and kept overnight before being filtered; the filtrate was made up to 50 mL.

##### Estimation

Contents of total soluble carbohydrates were determined using the anthrone technique according to Hansen and Møller (1975). The developed color was measured using an electric colorimeter at 620 nm. A blank mixture containing distilled water and reagent was used to set up the apparatus at zero optical density.

#### Total water-soluble proteins

##### Extraction

In this regard, one gram of air-dried *H. erinaceus* fruiting bodies was extracted (in a 250 mL conical flask) at 60 °C using a mixture of 10 mL distilled water and 5 mL of 2% phenol solution. The contents of the flasks were shaken well and kept overnight before being filtered, and then they were used for the estimation of soluble proteins (Mohd Rosni et al. 2015).

##### Determination

This assay was accomplished by the method described by Lowry’s method (Waterborg 2009). The optical density of the resulted color was then read at the wavelength of 750 nm. The concentration of soluble protein present in the sample was then calculated by making use of the constructed standard curve of proteins.

### Extraction of the bioactive crude extract by different solvents

The powder of fruiting bodies of *H. erinaceus* was extracted by 6 different solvents with a variable polar gradient: water, ethanol, acetonitrile, acetone, chloroform, and ethyl acetate. A known constant ratio of 10/100 (w/v) was used for this extraction protocol. All 6 extracts had been passed to estimate their antioxidant, antimicrobial, antiviral, and anti-inflammatory activities.

### Antioxidant assay

The antioxidant activity of the crude extract was determined by the DPPH free radical scavenging assay in triplicate and average values were considered. According to (Yen et al. 2002), a freshly prepared (0.004% w/v) methanol solution of 2,2-diphenyl-1-picrylhydrazyl (DPPH) radical was prepared and stored at 10 °C in the dark. A methanol solution of the test compound was prepared. A 40 µL aliquot of the methanol solution was added to 3 mL of DPPH solution. Absorbance measurements were recorded immediately with a UV–visible spectrophotometer (Milton Roy, Spectronic 1201). The decrease in absorbance at 515 nm was determined continuously, with data being recorded at 1 min intervals until the absorbance stabilized (16 min). This assay was proceeded in corresponding to ascorbic acid as a reference standard (Ali et al. 2022).

### Anti-inflammatory assay

Screening for the anti-inflammatory activity was carried out by using the simple economical assay: in vitro inhibition of albumin denaturation technique (Rahman et al. 2015) in corresponding to sodium diclofenac as a positive control (50 µg/mL).

### Antimicrobial assay

Agar well diffusion method was applied to determine the antimicrobial activity of five extracts against 6 pathogenic microorganisms; the first two belonged to Gram-positive bacteria (MRSA and *Streptococcus mutans*), the next two belonged to Gram-negative bacteria (*Enterobacter cloaca* and *Salmonella typhimurium*), and the last one is *Candida lipolytica* as a yeast species. This assay was done in corresponding to gentamycin as a positive control antibacterial and ketoconazole as a positive control antifungal (Kamel et al. 2022; El-Naggar et al. 2022).

### Antiviral assay

Assessment of antiviral activities of the ethanolic extract of fennel was achieved stepwise by detecting the maximum non-toxic concentration (MNTC) of the extract on the Vero cell line (ATCC CCL-81™) by the same MTT protocol mentioned previously, while the second step dealing with testing the effect of MNTC against Hepatitis A virus (HAV). Antiviral activities assessment was performed as follows: transferring 200 µL media into each well in a 96 well ELISA plate, adding 10^4^ cells corresponding to blank and control; incubation at 37 °C, 5% CO_2_ overnight; mixing of MNTC and viral suspension in a ratio of 1:1; and incubating this mixture for 1 h, adding 100 µL of this mixture into the wells, shaking at 150 rpm for 5 min, and incubating at 37 °C, 5% CO_2_ for 24 h. 20 µL MTT solution was added to each well, shaking at 150 rpm for 5 min, incubating at 37 °C, 5% CO_2_ for up to 5 h. Throwing out the fluids by a clean towel, formazan was resuspended in 200 µL DMSO, shaking at 150 rpm for 5 min, and optical density was read at 560 nm (Suleiman and Helal 2022). All data were obtained by measuring the mean through three replicates, and the elevated values were cleansed.

### Chromatographic purification of active metabolites

All six extracts were air-dried and their residues were then resuspended in methanol to be ready for applying onto thin layer chromatography (TLC) plate (10 × 10 cm Merck aluminum sheet, silica gel 60, layer thickness 0.2 mm). Chromatographic bands were examined by a fully automated HPTLC instrument (CAMAG––Switzerland). The first unit is an automatic applicator (Linomate5) by which the crude extracts were loaded onto a TLC plate in a form of bands against Griseofulvin as an authentic marker. TLC plate was then transferred into an automated development chamber (ADC2 CAMAG) using toluene–ethyl acetate–formic acid 5:4:1 (v/v/v) as an elution buffer, followed by UV scanning via UV chamber unit at visible light, long UV wavelength (365 nm), short UV wavelength (254 nm), and long UV wavelength (365 nm). TLC plate was then sprayed with *p*-anisaldehyde 0.5% in (Conc. H_2_SO_4_–acetic acid–acetone 5:10:85) and then oven heated at (105 °C) for 10 min. Data obtained (color, R*f*_,_ and the shape of bands) were analyzed according to Paterson and Bridge (1994).

### GC–MS for prediction of subcomponents

Both water and ethyl acetate extracts that exhibited higher activity were firstly dried in the air under aseptic conditions and then analyzed by gas chromatography GC–MS (Shimadzu 2010 system, Japan) using RTX-2330 (fused silica) 30 m capillary column of 0.25 µm internal diameter and df (µm) 0.20 µm. The column was operated at an initial temperature of 160–250 °C at the rate of 5 °C /min. and was held for 30 min. The injector and detector temperatures were 240 °C and 250 °C, respectively. Carrier gas (nitrogen) was supplied at a total flow rate of 50 mL/min with a split ratio of 20:0 and subcomponents were identified by comparison with linked library (Sigma) (Abdel-Razek et al. 2020).

## Results and discussion

### Phytochemical screening

Phytochemical screening (Table 1) refers to a high content of flavonoids and phenolics with high percentage of protein and nitrogen; on the other hand, moderate amounts of tannins and saponins were determined as well as the presence of little amounts of carbohydrates, alkaloids, and oils. It is suggested that phenolic and flavonoid compounds have inhibitory effects on mutagenesis and carcinogenesis in humans when up to 1.0 g are ingested daily from a diet rich in fruits and vegetables (Mujić et al. 2011).

**Table 1:** Quantitative assay of *H. erinaceus* powder for its phytochemical compounds

Test	Value
Total phenolic acid (mg eq./gm gallic acid)	248.8
Total flavonoids (mg eq./gm rutin)	309.8
Total carbohydrate (mg/g)	1.83
Total nitrogen (mg/g)	21.96
Total protein (mg/g)	14.64
Total alkaloids (mg/g)	0.0121
Total tannins (mg/g)	0.043
Total saponins (mg/g)	0.021
Total oil (mg/g)	0.002

In addition, *H. erinaceus* mushroom showed a high nutritional value due to the presence of fats, proteins, crude fibers, and carbohydrates as well as high content of folic acid, flavonoids, phenolics compounds, tannins, and saponins (Egwim et al. 2011). A Mixture of *Ginkgo biloba* L. Leaf and *H. erinaceus* fruit extract was phytochemically evaluated and proved the existence of some active phytochemical compounds that attenuate scopolamine-induced memory impairments in mice (Hong et al. 2022).

### Estimation of the antioxidant activities of the crude extracts

Table 2 reveals that the aquatic extract showed the highest potentiality as an antioxidant followed by chloroform and ethyl acetate extracts. While acetonitrile extract had the least antioxidant activity. On the other side, ascorbic acid as a positive control exhibited 27.2 µg/mL as IC_50_.

**Table 2:** Scavenging activity of *H. erinaceus* extracts with different solvents according to DPPH technique

Extracts	Relative polarity gradient	IC_50_ (µg/mL)
Ascorbic acid	Positive control	27.2
Water	1	35.7
Ethanol	0.654	399.3
Acetonitrile	0.46	490.8
Acetone	0.355	396
Chloroform	0.259	206.8
Ethyl acetate	0.228	284.8

These results demonstrated that the *H. erinaceus* extracts can be used to overcome oxidative stress where the excess of reactive oxygen species (ROS) showed significant effects on human health including metabolic disease, heart disease, and cancer (Chandrasekaran et al. 2016). In this study, the aquatic *H. erinaceus* extract showed a high antioxidant effect allowing using this extract as a treatment against the harmful effect of free radical toxicity as an exogenous antioxidant defense to overcome the insufficient endogenous antioxidant defense system to prevent damage and risk of oxidative stress completely (Simioni et al. 2018).

In addition, it was reported that water extract of *H erinaceus* polysaccharides extracted by water display strong antioxidant activity and can decrease ischemia reperfusion induced by oxidative injury in experimental animals’ kidneys (Han et al. 2013). Also, *H.* *erinaceus* is a good source of exogenous antioxidants that has been traditionally used in China for the treatment of oxidative stress-associated disease (Jiang et al. 2014). The anhydrous ethanol extracts of *H.* *erinaceus* were reported to have significant levels of antioxidant compounds with strong reducing power, high scavenging rates against DPPH and superoxide anion-free radicals, and high inhibition rates on lipid peroxidation (Jiang et al. 2016).

### Assessment of anti-inflammatory activities of the crude extracts

Table 3 shows low anti-inflammatory activity of *H. erinaceus* extracts, in which the percentage of protein inhibition ranged from 0% to 6.55% against 68.47% as an inhibition percentage of the positive control (sodium diclofenac), where acetonitrile extract showed no effect, while ethyl acetate extract showed the highest inhibition with 6.55%. In contrast, several mushroom species have been studied for anti-inflammatory activity (Zuo et al. 2021). Also, *H. erinaceus* mycelium could act as an anti-inflammatory agent to bring about neuroprotection, including the prevention of ischemic injury to neurons (Lee et al. 2014). Anti-inflammatory activity of n-hexane, chloroform, ethyl acetate, and methanol extracts of mycelia in submerged culture from 5 commercially available medicinal mushrooms, namely, *Coprinus sinensis, Cordyceps mortierella, Hericium erinaceus, G. lucidum,* and *Armillaria mellea,* indicated that theses extracts from medicinal mushrooms exhibited anti-inflammatory activity that might be attributable to the inhibition of nitrous oxide (NO) generation and can therefore be considered a useful therapeutic and preventive approach to various inflammation-related diseases (Elkhateeb et al. 2019). Our results showed low anti-inflammatory effect ranged between 0 and 6.5%; this due to the method used in the extraction may affect the anti-inflammatory compound in the *H. erinaceus* which when compared with other scientific paper showed higher anti-inflammatory activity.

**Table 3:** Assessment of anti-inflammatory activity of *H. erinaceus* extracts

Extract	Conc. (µg/mL)	Abs. (255 nm)	Inhibition of protein denaturation (%)
Negative control (DMSO)	–	1.497	–
Water	500	1.433	4.28
Ethanol	400	1.467	2
Acetone	500	1.484	0.87
Acetonitrile	200	1.502	0
Chloroform	300	1.446	3.41
Ethyl acetate	300	1.399	6.55
Diclofenac Na	50	0.472	68.47

### Evaluation of the antimicrobial activities of the crude extracts

All six extracts were tested for their antimicrobial activities against five different species of indicator microorganisms as listed in Table 4. *Enterobacter cloaca* is the most susceptible bacteria followed by *Streptococcus mutans* then *Salmonella typhimurium*. MRSA is the most resistant to all extracts except acetone extract had a weak effect on it. Yeast also had a significant resistance for all investigated mushroom extracts excluding acetone and ethyl acetate that possessed moderate antifungal activity according to CLSI guidelines (Humphries et al. 2018).

**Table 4:** Evaluation of the antagonistic effect of the different mushroom extracts against the indicator microorganisms

Test organism	Inhibition zone diameter (mm)					
	Water	Ethanol	Acetonitrile	Acetone	Chloroform	Ethyl acetate
Gram-negative bacterium Gentamycin 4 µg (positive control)						
*Enterobacter cloaca*	15	15	12	13	**17**	16
* S. typhimurium*	10	–	9	–	11	**12**
Gram-positive bacterium Gentamycin 4 µg (positive control)						
MRSA	–	–	–	8	–	–
* S. mutans*	–	8	10	9	11	**12**
Yeast Ketoconazole 100 µg (positive control)						
* Candida lipolytica*	–	–	–	9	–	**10**

Ethyl acetate extract relatively had the most effective antimicrobial activity followed by acetone, chloroform, and acetonitrile, all of which belong to non-polar solvents, while water and ethanol which represent the polar solvents had not only a very narrow-spectrum antimicrobial activity but also their effects ranged from weak to moderate. This result is contrary to results reported by Sridhar et al. (2011) that methanol and aqueous extract of mushroom fruit bodies showed high antimicrobial activity against *Salmonella typhi* and *S. aureus*. Also, Jonathan and Awotona (2010) reported that in vitro antagonistic effect of the ethanol, methanol, and water extracts of *G. lucidum, G. applanatum,* and* G. australe* against some pathogenic microorganisms. There are few reports available on the possible use of *H. erinaceus* for the management of diseases. Therefore, in the present investigation, *H. erinaceus* was evaluated for its antimicrobial potential.

In contrast, studies on the antimicrobial activity of *P. ostreatus* using solvents with different polarities found that non-polar solvents, like petroleum ether extracts of *P. ostreatus* had a stronger inhibition activity on both Gram-positive and Gram-negative bacteria but with varying degrees of intensity (Pauliuc and Botau 2013). Also, Nehra et al. (2012) found that organic solvents consistently displayed better antimicrobial activity than the aqueous extract.

In this study, values of bacterial and fungal growth inhibition by the different mushrooms’ extracts were variable. This may be due to the use of different solvents and test microorganisms. The changeable antimicrobial activity of different extracts may be indicating the presence of different broad-spectrum antimicrobial compounds in the mushroom. Similar reports by other researchers return the variable of antimicrobial activity of mushroom extracts may arise from the genetic structure of mushroom species, physical, biochemical constituents, chemical differences of mushroom extracts, solvents, and test microorganisms that used when its antimicrobial properties compared to the other mushroom species (Shah et al. 2014; Smolskaitė et al. 2015).

### Antiviral activity determination

This experiment was performed in two steps; the first was designed to determine the maximum non-toxic concentration of tested extracts against a normal Vero cell line. This step showed that aquatic and ethyl acetate extracts exhibited no toxicity starting from 80 to 4.88 µg/mL, respectively, where aquatic extract showed 50% cytotoxic concentration CC_50_ of 111 µg/mL, while ethyl acetate extract showed CC_50_ of 26 µg/mL, which indicated that ethyl acetate has less toxicity than water extract against Vero cell line (Table 5). The second step involved the determination of IC_50_ for both non-toxic concentrations of water and ethyl acetate extracts (Table 6).

**Table 5:** Detection of maximum non-toxic concentration of the mushroom extracts against Vero cell (cytotoxicity)

ID	Conc. (µg/mL)	Mean OD	Viability %	Toxicity %	SE	CC_50_
Vero		0.132	100	0	0.009	µg/mL
Ethyl acetate extract	2500	0.014	10.6	89.4	0.003	26
	1250	0.0153	11.6	88.4	0.0003	
	625	0.0147	11	89	0.0008	
	312.5	0.015	11.3	88.7	0.001	
	156.25	0.0167	12.6	87.4	0.0007	
	78.12	0.014	10.6	89.4	0.0005	
	39.06	0.0293	22.2	77.8	0.0024	
	19.53	0.0883	67	33	0.0013	
	9.76	0.1037	78.5	21.5	0.0034	
	4.88	0.125	99.7	0.3	0.0051	
	2.44	0.131	99.8	0.2	0.0015	
	1.22	0.134	101.5	0	0.002	
Water extract	1280	0.0133	10	90	0.0003	111
	640	0.015	11.4	88.6	0.0021	
	320	0.0207	15.7	84.3	0.000882	
	160	0.037	28	72	0.002517	
	80	0.122	92.4	7.6	0.003606	
	40	0.1327	100	0	0.001202	
	20	0.133	101	0	0.002186	
	10	0.133	101	0	0.000333

**Table 6:** Detection of antiviral activity for MNTC of mushroom extracts

Test	Conc (µg/mL)	Mean O. D	Viability %	Toxicity %	Viral activity%	Antiviral effect %	IC_50_ µg/mL
Vero		0.132	100	0			
HAV		0.0887	67.2	32.8	100	0	
Ethyl acetate	4.88	0.0877	66.4	33.6	102.3	0	
Water	80	0.13	98.5	1.5	4.6	95.4	25
	40	0.1257	95	5	14.6	85.4	
	20	0.105	79.5	20.5	62.3	37.7	
	10	0.087	66	34	103.8	0	

Table 6 shows that the non-toxic concentrations of ethyl acetate had no antiviral activity against HAV in vitro, but water extract had a good opportunity to be a useful agent with antiviral activity and its IC_50_ is 25 µg/mL which is completely non-toxic for a normal cell line that reflects safety use.

These results demonstrated the ability of *H. erinaceus* extracts to be used as a source for antiviral drugs and it has resembled many researchers showed the ability of mushroom extracts to have an antiviral effect (Seo and Choi 2021). It was reported that both aqueous and ethanol extracts of *L. edodes* showed a high antiviral activity on the replication of poliovirus type 1 and bovine herpesvirus type 1 (Rincão et al. 2012). Also, lentinan was purified and showed antiviral activity from shiitake *Lentinula edodes,* which can suppress the surface expression of HIV (Seo and Choi 2021).

Furthermore, *H. erinaceus* aqueous extracts and other aqueous extracts of other mushrooms exhibited low toxicities on Vero cells with promising antiviral activities against DENV2 (Ellan et al. 2019); arboviruses were also antagonized by *H. erinaceus* extracts (Goh et al. 2020). Also, *H. erinaceus* inhibited the progression of HSV and HIV (Chun et al. 2021; Choengpanya et al. 2021).

### Prediction of the chemical composition of mushroom extracts using a fully automated TLC scanner

All six extracts of *H. erinaceus* were applied onto a TLC plate and allowed to flow using a certain mobile phase against Griseofulvin as authentic; the analyzed tracks were visualized by UV at two different wavelengths 254 and 365 nm, and all resulting peaks are reported with their Rf in Table 7. Table 7 reveals the presence of ten different compounds with different Rf values, and it is clear to observe that water extract is the least successful one to be separated by the selected mobile phase which only exhibited the presence of only three peaks followed by ethanolic extracts in which only 4 peaks were detected, while acetonitrile and acetone extracts exhibited the most successful separation followed by chloroform and ethyl acetate extracts which exhibited the presence of 6 and 5 peaks, respectively.

**Table 7:** Preliminary identification and distribution of the subcomponents of the crude extracts by a fully automated HPTTLC scanner

Seq	Rf	Mushroom extracts					
		Water	Ethanol	Acetonitrile	Acetone	Chloroform	Ethyl acetate
1	15	−	+	−	−	−	−
2	20	+	+	+	−	−	−
3	31	+	+	+	+	−	+
4	42	−	+	−	−	−	−
5	45	−	−	+	+	+	+
6	48	−	+	+	+	−	+
7	60	+	+	+	+	+	+
8	70	−	−	+	+	+	+
9	77	−	−	−	+	+	−
10	86	+	−	−	−	+	−

### GC–MS to preliminary identify mushroom extracts

As a result of antimicrobial and anti-inflammatory activities evaluation, ethyl acetate extract gave superior results to the other selected solvents. On the other hand, aquatic extract gave superior results as an antioxidant and antiviral active extract. Subsequently, those two extracts were selected to be analyzed by GC–MS as an attempt to preliminary identify their subcomponents in addition to the results of HPTLC.

Table 8 reveals the presence of a total of 16 different compounds, 13 out of 16 belong to ethyl acetate extract, while 6 out of 16 belong to aquatic extract. Both Quercetin/derivative and Lucenin-2 are common in both extracts, both are considered important flavonol (flavonoid compounds), and also they have a wide spectrum of biological activities as antibacterial, antiviral, anti-inflammatory, etc. (Kim and Park 2018; Kim et al. 2016a). As for the HPTLC results that revealed the presence of 10 different significant peaks, GC–MS exhibited a higher sensitivity that could detect 16 peaks in only two selected solvents. In addition, four and five peaks were detected by HPTLC for water and ethyl acetate extracts, respectively. While, GC–MS detected 6 and 13 peaks for water and ethyl acetate extracts, respectively.

**Table 8:** Preliminary identification and distribution of the subcomponents of the aquatic and ethyl acetate extracts by GC–MS

Compound predicted	RT (min)	MW	Molecular formula	Ethyl acetate	Area %	Water	Area %	Application
2-amino-3-phenyl-6-nitroindole	13.73	253	C_14_H_11_N_3_O_2_	−	0	+	18	Anaphylactic reactions in animals (Grinev et al. 1983)
Quercetin	28.08	344	C_18_H_16_O_7_	+	12	+	10	Treatment of Gastrointestinal Cancers (Mirazimi et al. 2022)
1-hexadecanol,2-methyl	29.66	256	C_17_H_36_O	+	2.5	−	0	Antioxidant, antimicrobial, hemolytic (Ouyang et al. 2012)
1-Eicosanol	29.79	298	C_20_H_42_O	+	18	−	0	Antioxidant, antimicrobial (Chatterjee et al. 2018)
Quercetin-7,3,4-trimethoxy	30.21	344	C_18_H_16_O_7_	+	1	+	6	Antioxidant, antimicrobial (Materska 2008)
Isochiapin B	30.79	346	C_19_H_22_O_6_	+	1	−	0	Anti-insect, antimicrobial, antioxidant, and anticancer activities (Elkhateeb et al. 2020)
17-pentatriacetontene	32.08	490	C_35_H_70_	+	1	−	0	Anti-inflammatory Anticancer Antibacterial Antiarthritic (Kumar et al. 2018)
Oleic acid ester	32.96	610	C_39_H_76_O_3_	+	3	−	0	Antioxidant, antiproliferative (Elagbar et al. 2016)
Lucenin-2	33.01	610	C_27_H_30_O_16_	+	2	+	7	Anti-inflammatory (Kim et al. 2016b)
10-Nonadecanone	35.97	282	C_19_H_38_O	+	6	−	0	Not available
Hexadecanoic acid, methyl ester	36.98	270	C_17_H_34_O_2_	+	7	−	0	Antioxidants, hypocholesterolemia, nematicide, and pesticide (Siswadi and Saragih 2021)
1-Eicosanol	38.28	298	C_20_H_42_O	+	11	−	0	Antibacterial, antitumor (Chatterjee et al. 2018)
1-Tricosanol	41.90	340	C_23_H_48_O	+	5	−	0	Antimicrobial (Tayade et al. 2013)
Oleic acid, eicosyl ester	42.64	562	C_38_H_74_O_2_	−	0	+	2	Larvicidal (Gurunathan et al. 2016)
Total	14			12		5		

Water extract of *H. erinaceus* contained 5-Methyldelphinidin (pulchellidin) is considered as one of the methylated anthocyanidins which exert some of their activities through their binding to the plasma membrane receptors and activating important signaling pathways without entering the cell (Jiménez et al. 2010). The rest of the other subcomponents belong to long-chain hydrocarbons, alcohols, oleic acid ester which is considered a polyunsaturated fatty acid PUFA (Hashem et al. 2022), and isochiapin B which is considered an essential oil (Değirmenci and Erkurt 2020).

## Conclusion

The discovery of new antimicrobial compounds is therefore becoming ever important. The fact that edible mushrooms are non-toxic and contain various compounds beneficial to human health, encouraged us to study the mushroom’s activities as a source of antimicrobial drugs. *H. erinaceus* crude extracts showed a noticeable effect against the tested microorganisms, including MRSA, and *Streptococcus mutans*, *Enterobacter cloaca*, *Salmonella typhimurium*, and *Candida lipolytica,* demonstrated the ability of using theses extracts as antimicrobial agent topically or orally and may have synergetic effect when combined with the commercial antibiotics to overcome the drug resistance bacteria; this needs further investigation and testing in combination with other drugs. Also, promising antioxidant and antiviral activities of the aquatic mushroom extract encourage us to recommend using this mushroom as antioxidant and antiviral supplements. Thus, *H. erinaceus* could represent an essential part of human meals every day to protect him against either oxidative stress or viral attack. The highly nutritious and antioxidant values of *H. erinaceus* may also be potentially an added benefit to the patients. In addition, GC–MS analysis reported the presence of different components with variable biological activities, for example, quercetin/derivative and Lucenin-2 in the *H. erinaceus* extracts demonstrated the ability of theses extracts to be used for medical treatment of inflammation, and bacterial and viral infection as they both considered flavonoid compounds and have antibacterial, antiviral, anti-inflammatory effect. More studies will be needed to isolate and identify the pure active compounds as well as determination of the mode of action of these antimicrobial compounds, we believe that it is worthwhile to exploit the potential of these antimicrobial compounds in treating the infectious bacterial and fungal diseases.

## Data Availability

All data generated or analyzed during this study are included in this published article.
